# Biotechnology-derived drugs: how far has Morocco come?

**DOI:** 10.11604/pamj.2022.43.159.37978

**Published:** 2022-11-28

**Authors:** Sanaa Zaoui, Amal Habchane, Soukaina Khatem, Ayyoub Alioua

**Affiliations:** 1 Department of Pharmacology and Toxicology, Clinical Research Center, Mohammed VI University Hospital, Marrakesh, Morocco,; 2 Faculty of Medicine and Pharmacy, Bioscience and Health Laboratory, Cadi Ayyad University, Marrakesh, Morocco,; 3 Faculty of Medicine and Pharmacy, Science and Technology and Medical Sciences, Bioscience and Health Laboratory, Cadi Ayyad University, Marrakesh, Morocco

**Keywords:** Biological drug, biosimilar, Morocco, marketing, regulation

## To the editors of the Pan African Medical Journal

Thanks to advances in biotechnology, millions of people have been treated for many serious and rare diseases [1]. A biological drug is a product where the active ingredient is a biological substance produced or extracted from a biological source, and for which the characterization and determination of quality requires a combination of physicochemical and biological tests as well as knowledge of its development process and control [2]. When the patents protecting these drugs expire, biosimilar drugs can be developed by other biotechnology manufacturers, as is the case for generic drugs. This helps to reduce costs for patients and social security systems. A biosimilar drug is a biological drug with the same active substance composition, both qualitatively and quantitatively, and the same pharmaceutical form as the reference biological drug. A copy of a biological molecule cannot be strictly identical but only similar to the reference product. This is particularly due to differences related to the variability of the raw material or the manufacturing processes. A biosimilar also requires the production of additional preclinical and clinical data under conditions determined by regulation [3]. In Morocco, decree No 2-14-841 published on August 5^th^, 2015, and related to the marketing authorization of drugs for human use, has set for the first time a regulatory framework for new pharmaceutical products, such as biological drugs and their biosimilars. It has regulated the administrative procedure for the marketing authorization of biosimilars. This new decree and a circular published a year later have set specific requirements for the registration of biosimilars and defined the characteristics of the registration application files and the elements of submission. They also have brought several improvements leading to more transparency and equality in terms of the administrative treatment of the files submitted by pharmaceutical laboratories. In addition to this administrative framework, there were special and rigorous requirements, especially regarding the comparability studies of the raw material, the manufacturing processes, and the preclinical and clinical trials with the reference drug, which must meet the standards and guidelines of the World Health Organization concerning biosimilar drugs.

The risk management plan was one of the new regulatory requirements. Henceforth, the manufacturing company must submit a risk management plan to health authorities to launch a new biosimilar on the market. This plan must outline the measures to be taken to minimize risks and anticipate any adverse effects [4]. The biological drugs available on the Moroccan market belong to several classes, including antineoplastics, immunomodulators, antithrombotics, hormones, erythrocyte growth factors, and immunoglobulins. The health insurance system covers most of the currently commercialized drugs (Table 1). In order to construct this table, we have searched for each of the drugs mentioned in it containing the mentioned substance on a national database containing drugs available in Morocco [5]. We then searched their coverage status on the “Caisse Nationale des Organismes de Prévoyance Sociale” (CNOPS) website [6]. If the status wasn’t available on this website, we would search for it on another database [7]. Concerning biosimilars, the pharmaceutical industry's turnover recorded an increase from 5 million dirhams to 452 million dirhams from 2015 to 2019. In terms of quantity, the number of produced packs has increased from 84000 to 772000 units over the same period. Sixty-three biological drugs have obtained marketing authorizations from the Health Ministry from 2016 to 2018, including 6 biosimilars. The recommendations of the competition council regarding the situation of competition in the pharmaceutical market in Morocco include taking budgetary, regulatory, and administrative incentive measures, to encourage the local production of drugs, including biosimilars [8]. Since then, investments in the field of biotechnology have been carried out in Morocco to make it possible to locally produce biological drugs. In 2019, a Moroccan pharmaceutical company celebrated the inauguration of its biotechnology-based anticancer unit. As a result, the cost of one course of treatment of an anticancer drug has become four times less expensive [9]. In 2022, the foundation stone of the future mega factory for manufacturing and syringing vaccines was laid. This project is worth 500 million euros and aims to create an African biopharmaceutical and vaccine innovation hub in Morocco with global recognition. In the first phase, Morocco plans to launch the production of test batches as early as July 30^th^, 2022. The second phase should cover, in less than 3 years, more than 70% of the national needs and more than 60% of those of the continent, by transferring the aseptic filling and manufacturing active substances of more than 20 vaccines and biotherapeutic products [10].

**Table 1:** list of biological drugs available in Morocco and their coverage by the health insurance system

Therapeutic class	Active substance	Reference biological drug		Biosimilar drug	
		Brand name	Coverage	Brand name	Coverage
Antineoplastic and immunomodulating agents	Adalimumab	Humira™	Yes*	Amgevita™	Yes*
	Bevacizumab	Avastin™	Yes	Ypeva™	Yes
	Etanercept	Enbrel™	Yes		
	Infliximab	Remicade™	Yes	Remsima™	Yes
	Rituximab	Mabthera™	Yes	Zelva™	Yes
	Pegfilgrastim	Neulastim™	Yes*		
	Filgrastim	Neupogen™	Yes	Nivestim™	Yes*
				Zarzio™	Yes
	Trastuzumab	Herceptin™	Yes	Herzuma™	Yes
		Kadcyla™	No	Kanjinti™	No
				Hertraz™	Yes
				Trazuva™	Yes
Antithrombotic agents	Enoxaparin	Lovenox™	Yes	Flumax™	Yes
				Novex™	Yes
Sex hormones and modulators of the genital system	Follitropin Alfa	Gonal-f ™	Yes		
Systemic hormonal preparations	Somatropin	Genotropine™	Yes	Omnitrope™	Yes
		Norditropine™	Yes*		
		Umatrope™	Yes		
		Saizen Click Eazy™	No		
Antianemic preparations, erythrocyte growth factors	Epoetin	Eprex™	Yes*	Binocrit™	Yes*
		Recormon™	Yes*	Epotin™	Yes
		Aranesp™	Yes	Hemax™	Yes
				Potex™	Yes
Insulins and analogues	Insulins	Actrapid™	Yes	Basalog™	Yes
		Apidra™	Yes*	Insulet™	Yes
		Humalog™	Yes*	Biosulin™	Yes
		Insulatard™	Yes		
		Lantus™	Yes*		
		Levemir™	Yes*		
		Mixtard™	Yes		
		Novomix™	Yes		
		Novorapid™	Yes		
		Toujeo™	No		
		Umuline™	Yes		
Immunoglobulins	Normal human immunoglobulin	Tegeline™	Yes		
		Clairyg™	Yes		
		Immunoglobuline normale IV LFB-CNTS™	Yes		
		Gammanorm™			
		IG Vena™	No		
		Octagam™	No		
			Yes*		
	Human anti-D immunoglobulin	Rhesonativ™	Yes	Immunorho™	Yes
				Natead™	Yes
				Rhophylac™	Yes
	Human hepatitis B immunoglobulin	Immuno HBs™	No		
	Tetanus immunoglobulin	Antitoxine™	Yes*		

* Not covered in some forms

## Conclusion

In Morocco, industrial companies must be encouraged to locally produce biomedicines. This would allow for covering part of the national needs and reducing costs, which will increase patients' access to such drugs.
